# *In Vivo* Fate Analysis Reveals the Multipotent and Self-Renewal Features of Embryonic *AspM* Expressing Cells

**DOI:** 10.1371/journal.pone.0019419

**Published:** 2011-04-29

**Authors:** Cinzia Marinaro, Erica Butti, Andrea Bergamaschi, Alessandro Papale, Roberto Furlan, Giancarlo Comi, Gianvito Martino, Luca Muzio

**Affiliations:** 1 Department of Neuroscience, Institute of Experimental Neurology (INSPE)-San Raffaele Institute, Milan, Italy; 2 Department of Neurology, Institute of Experimental Neurology (INSPE)-San Raffaele Institute, Milan, Italy; Instituto de Medicina Molecular, Portugal

## Abstract

Radial Glia (RG) cells constitute the major population of neural progenitors of the mouse developing brain. These cells are located in the ventricular zone (VZ) of the cerebral cortex and during neurogenesis they support the generation of cortical neurons. Later on, during brain maturation, RG cells give raise to glial cells and supply the adult mouse brain of Neural Stem Cells (NSC). Here we used a novel transgenic mouse line expressing the CreER^T2^ under the control of AspM promoter to monitor the progeny of an early cohort of RG cells during neurogenesis and in the post natal brain. Long term fate mapping experiments demonstrated that AspM-expressing RG cells are multi-potent, as they can generate neurons, astrocytes and oligodendrocytes of the adult mouse brain. Furthermore, AspM descendants give also rise to proliferating progenitors in germinal niches of both developing and post natal brains. In the latter –i.e. the Sub Ventricular Zone- AspM descendants acquired several feature of neural stem cells, including the capability to generate neurospheres in vitro. We also performed the selective killing of these early progenitors by using a Nestin-GFP^flox^-TK allele. The forebrain specific loss of early AspM expressing cells caused the elimination of most of the proliferating cells of brain, a severe derangement of the ventricular zone architecture, and the impairment of the cortical lamination. We further demonstrated that AspM is expressed by proliferating cells of the adult mouse SVZ that can generate neuroblasts fated to become olfactory bulb neurons.

## Introduction

Radial Glia cells (RGs) derive from neuroepithelial cells of the early embryos. At the onset of neurogenesis, RG cells perform symmetric and asymmetric division to support either the proliferating pool of cells or the generation of cortical plate neurons. RG cells are also fated to become glial cells in the post natal brain and a subset of them is maintained and propagated in specialized germinal/neurogenic niches of the adult brain, as neural stem cells (NSCs) [1]. NSCs are located in the sub ventricular zone (SVZ) of the lateral ventricle and in the dentate gyrus of the hippocampus [2], [3]. SVZ-restricted NSCs cells generate fast cycling precursors –i.e. type-C progenitors- that subsequently give raise to neural precursor cells (type-A, neuroblasts), fated to become olfactory bulb neurons [2]. NSCs, however, are multipotent cells, because *in vitro* [4] and *in vivo* they can generate, neurons, oligodendrocytes and astrocytes of the adult brain [5], [6], [7], [8].

During neurogenesis the balance of asymmetric versus symmetric divisions regulates the number of post-mitotic cells and, possibly fosters the maintenance of a proliferating pool of cells fated to supply neural stem cell niches of proliferating progenitors. The identification of these cells in both developing and adult brains, has been obtained by using feasible genetic tools that have so far been generated to label RG and SVZ restricted NSCs [9], [10], [11], [12], [13], [14]. These models give the opportunity to trace, *in vivo*, SVZ restricted NSCs expressing self renew features [14], and to perform high resolution long term fate mapping analysis by targeting proliferating cells during forebrain development. Based on these models, it has been established that cohorts of genetically tagged embryonic precursor cells can generate SVZ restricted NSCs [1], [11], [14]. The analysis of a battery of Cre transgenic mouse lines revealed that SVZ restricted NSCs derive from both cortical RG cells and cells of lateral Ganglionic Eminence [15], [16].

Accordingly to this view, genes capable to regulate the balance between symmetric and asymmetric cell division may influence embryonic neurogenesis and, above all, they can extinguish/increase the number of cells fated to acquire features of long term proliferating elements. Indeed, defects in cell division occurring in the developing brain can generate microcephaly in human. The deviation from the normal brain development has been associated to a severe reduction of brain size and cognitive defects [17]. The product of *Abnormal Spindle-like Microcephaly associated* gene (*AspM*) is an interesting candidate to control the balance of symmetric and asymmetric cell divisions. Indeed, several experimental evidence suggests that *AspM* can regulate the positioning of the mitotic spindle during neuroblast cell division [18] and mutations in *ASPM* represent a leading cause of primary microcephaly, possibly interfering with cell proliferation/differentiation equilibrium of cortical neuroblasts [19], [20]. Furthermore, ASPM is highly expressed in glioblastomas. In this cells, as well as in NSCs, the inhibition of ASPM dramatically affects their *in vitro* cell cycle progression [21]. The inactivation of *AspM* in transgenic mice leads to a significant reduction of the brain size and a massive reduction of germ cells [22]. Altogether these data highlight the potential role of *AspM* in controlling self-renewal of RG cells, and put forward the intriguing hypothesis that a cohort of *AspM*-expressing cells might foster the maintenance of symmetrically dividing long term progenitors. However, functional experiments supporting this hypothesis are still missing.

Here we used a novel AspMCreER^T2^ inducible allele to perform *in-vivo* long term fate mapping of a relative earlier cohort of embryonic *AspM*-expressing cells during neurogenesis. Cre mediated recombination was induced at the beginning of neurogenesis in a selected cohort of proliferating RG cells that were subsequently traced during forebrain development and in post natal neurogenic niches. This cohort of early *AspM*-expressing cells give raise to neurons, astrocytes and oligodendrocytes of the mature brain. In addition, a subset of them was maintained in germinal niches of the post natal brain as proliferating progenitors that express several molecular features in common with aNSCs, including self renewal. We next extended fate mapping experiments to the adult brain by changing the activation paradigm of the AspMCreER^T2^ allele. We next tested the contribution of embryonic *AspM*-expressing cells to the maintenance of proliferating pools of the brain, by the selective killing of these cells.

## Results

### AspM-CreER^T2^ transgene targets Cre-mediated recombination in long term proliferating cells of the cerebral cortex

In order to trace the progeny of early *AspM* expressing cells, we generated a transgenic mouse line, in which the inducible *Cre* recombinase (CreER^T2^) [23], [24], [25] -i.e. transiently activated by injecting mice with Tamoxifen (Tam) [26], [27]-, was placed under the control of *AspM* cis-acting regions (Figure S1A and material method section for a detailed description of the construct). *AspM* and *Cre* mRNA distributions, were compared on parallel coronal sections of both embryonic (E15.5) and adult (P30) AspMCreER^T2^ brains by radioactive *in-situ* hybridization. Both transcripts were expressed in the VZ [19] of the E15.5 brain (Figure S1B, C) and in cells of the post-natal SVZ (Figure S1D, E). RT-PCR experiments on microdissections of peri-ventricular regions of the lateral ventricle confirmed that both *AspM* and *Cre* mRNAs are expressed in the postnatal SVZ (Figure S1F).

**Figure 1:**
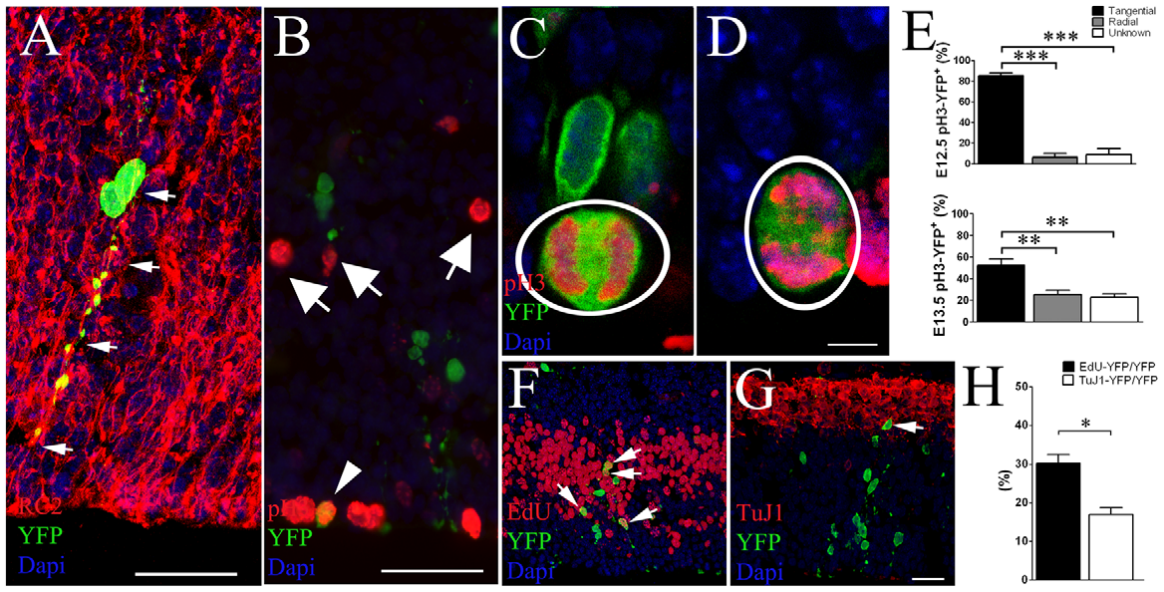
Cre mediated recombination of early AspM expressing cells. AspM-CreER^T2^/Rosa26YFP mice were injected with Tam at E11.5 and E12.5 and embryos collected at E13.5 (n = 4). A, Confocal micrographs of E13.5 cortical wall stained for RC2 and YFP. Arrows indicate double positive cells. B, E13.5 cortical wall stained for pH3 and YFP, arrows indicate pH3^+^ cells in the SVZ. Confocal images of E13.5 coronal sections stained for pH3 and YFP detection show cells with mitotic spindle oriented either along the horizontal plane (C) or the vertical plane (D). Panel E shows percentages (±S.D.) of YFP^+^ tangential and radial divisions counted in the ventricular lining of E12.5 and E13.5 brains (n = 4 for each group) that were pulsed with Tam at E10.5/E11.5 and E11.5/E12.5 respectively. One hour before sacrifice, E13.5 embryos received a single injection of EdU and sections were stained for YFP and EdU detection (arrows in F indicate double positive cells). The Percentage (± S.E.M.) of cells incorporating EdU is plotted on panel H. Parallel sections were also stained for YFP and TuJ1 (arrow in G shows a double positive cells in the outer cortical wall). The percentage (± S.E.M.) of TuJ1/YFP^+^ cells is plotted on histogram of the panel H. Scale bar 10 µm (C and D) and 40 µm (A, B, F and G). *** p<0.001, ** p<0.01, * p<0.05, t-student.

To analyze the recombination efficiency and to trace *AspM*-expressing cells during neurogenesis, we crossed mice carrying the AspMCreER^T2^ allele with mice carrying the *Cre*-dependent reporter allele Rosa26YFP [28], in which a floxed stop codon was cloned upstream YFP to allow genetic fate mapping. Double transgenic mice were injected twice with Tamoxifen (Tam) at E10.5/11.5 and brains were collected at E12.5 to study the expression of the YFP reporter after a short *Cre* induction. YFP^+^ cells were preferentially placed in germinal niches of injected brains. Within the VZ/SVZ, cluster of YFP^+^ cells also co-expressed the *AspM* mRNA (Figure S1G). Fifteen (±4.1)% of AspM expressing cells recombined the Rosa26YFP locus after Tam administration (Figure S1H). The expression of *AspM* and the Cre-mediated recombination of Rosa26YFP locus were also investigated in post natal brains. Although *AspM* is greatly reduced in adult brains [19], the Cre-mediated recombination of Rosa26YFP locus occurred in approximately 12% of SVZ-confined AspM^+^ cells of P30 double transgenic mice treated with Tam for 5 days (Figure S1I). AspMCreER^T2^/Rosa26YFP embryos were further treated with Tam at E11.5/E12.5, and brains were subsequently analyzed at E13.5. Cells expressing YFP were preferentially clustered in the germinal region of the cortical wall, the vast majority of them expressed the RG cell marker RC2 [29], (Figure 1A). Very few, if any, of them co-localized with SVZ mitotic cells expressing the phospho-Histone 3 (pH3) marker (Figure 1B). Tangential versus radial cell divisions were analyzed on E12.5 (pulsed with Tam at E10.5/11.5) and E13.5 (pulsed with Tam at E11.5/12.5) embryos, by staining sections for YFP and pH3 (Figure 1C, D). Accordingly to previous results [30], more than 80% of YFP^+^ mitosis displayed a cleavage plane perpendicularly oriented to the ventral surface (Figure 1E, upper panel). This number, however, slightly decreased at E13.5, possibly for the presence of many mitosis with unknown orientation (Figure 1E, lower panel). Accordingly, only 6.12% (±3.6) and 25.1% (±4.0) of pH3/YFP double positive mitosis exhibited an horizontal cleavage plane (radial oriented mitotic spindle) at E12.5 and E13.5 respectively. These results confirmed a previous report [18] that indicates the expression of *AspM* enriched in tangential mitosis (Figure 1E). E13.5 mice, pulsed with Tam at E11.5/E12.5, received a single injection of 5-ethynyl-2′-deoxyuridine (EdU) one hour before the sacrifice to label proliferating cells (Figure 1F). 30.2% (±2.2) of cortical YFP^+^ cells incorporated the EdU tracer (Figure 1H). On the other hand, parallel sections stained for TuJ1 and YFP (Figure 1G) showed that only 16.9% (±1.7) of YFP^+^ cells co-expressed the neuronal post mitotic marker TuJ1 (Figure 1H).

**Figure 2:**
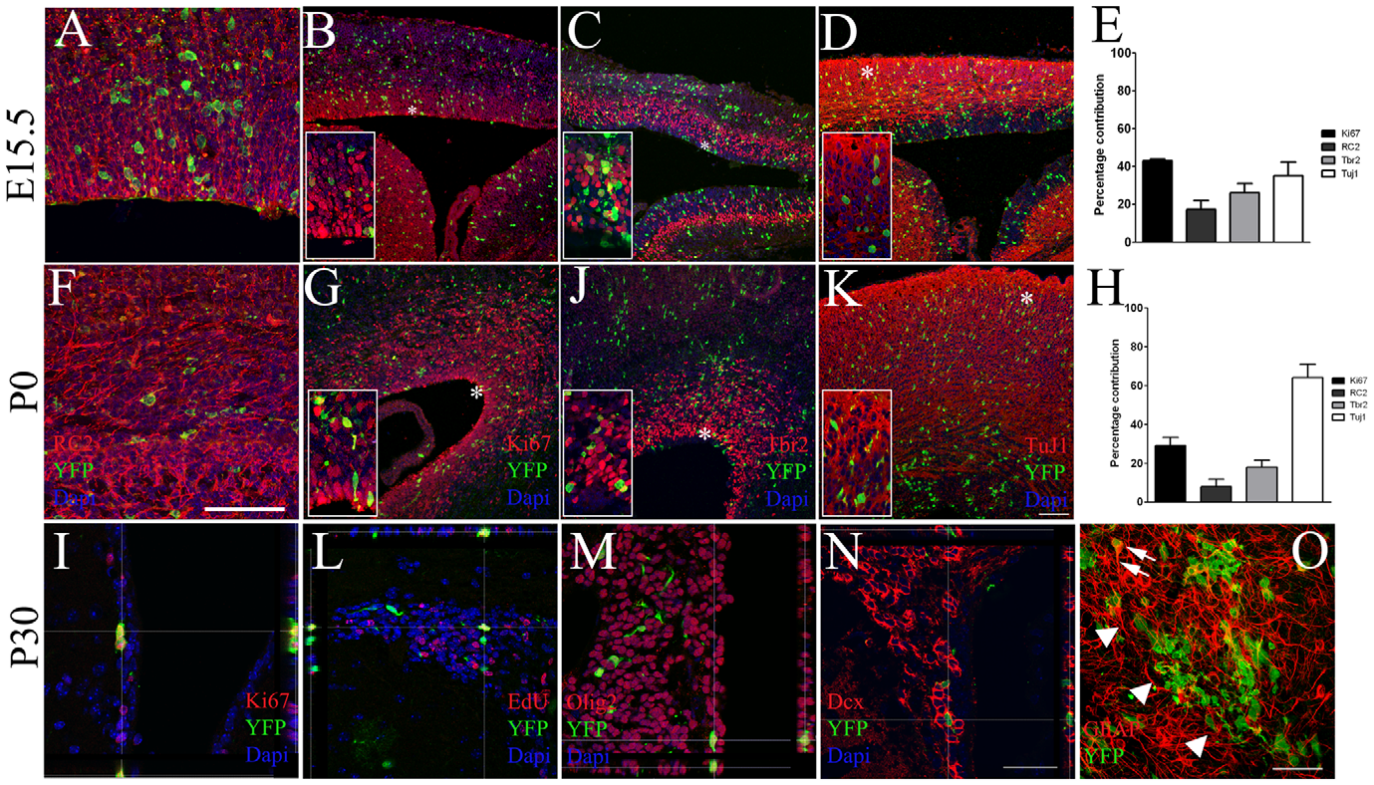
Embryonic AspM precursors generate cortical neurons and proliferating cells of embryonic and post natal germinal niches. AspM-CreER^T2^/Rosa26YFP mice were administered with Tam at E12.7 and E13.2 and then brains were collected at E15.5 (n = 4, A–E), P0 (n = 4, F–H) and P30 (n = 4, I–O). Confocal optic sectioning of brain coronal slices were probed for YFP and: RC2 (A, F), ki67 (B, G), Tbr2 (C, J) and TuJ1 (D, K). YFP^+^ cells expressing each marker were found within the cortical wall. Histograms in E and H show the mean percentages (± S.D.) of double positive cells counted at each time point from 4 independent experiments. High magnification images are provided in insets, asterisks indicate their position on low magnification pictures. P30 mice received two daily injections of EdU for 5 consecutive days before the sacrifice. SVZs were labeled for YFP and: ki67 (I), EdU (L), Olig2 (M) and Dcx (N). Whole mount of the lateral ventricle stained for YFP and GFAP is provided in panel O. Arrows indicate double positive cells and arrowheads a cluster of YFP^+^ cells. Scale bars 100 µm.

To perform long term fate mapping of *AspM* expressing cells, the AspM-CreER^T2^ allele was activated by Tam at E12.7 and E13.2 and brains collected at E15.5, P0, P30 and P90. At E15.5 and P0, a large number of YFP^+^ cells were located in the VZ/SVZ of double transgenic mice expressing markers of RG and basal progenitor (BP) cells. Indeed, 17.2% (±4.6) of YFP^+^ cells at E15.5 expressed RC2 (Figure 2A), 43% (±0.8), the proliferation marker Ki67 (Figure 2B) and 26% (±4.8) were Tbr2^+^, which is a marker of BP cells [31] (Figure 2C). At P0, the number of YFP^+^ cells co-expressing RC2 was slightly reduced (7.9±3.6%) (Figure 2F). Also the fractions of YFP^+^ cells displaying the proliferation markers (Ki67) and BP (Tbr2) were reduced to 29%(±4.1) and 17.9% (±3.5) respectively (Figure 2G, J). As expected, *AspM* descendants give raise to cortical plate neurons. Indeed, 35%(±7.3) of YFP^+^ cells co-expressed TuJ1 at E15.5 (Figure 2D). This number significantly increased at P0 to 64% (±6.9) (Figure 2K).

We next investigated the distribution of E12.7/E13.2 traced *AspM*-descendants in germinal niches of the post natal brain. YFP^+^ cells were still detected in the post natal germinal niches of the P30 brain. In the SVZ we found that 39.2%(±21.6) of YFP^+^ cells co-expressed the proliferating marker Ki67 (Figure 2I) and incorporated the S-phase tracer EdU (Figure 2L) [32]. YFP^+^ cells co-expressed also markers that are expressed by subsets of adult neural progenitor cells, such as: Olig2 [33] (Figure 1M), and the neuroblast markers Dcx and PSA-NCAM (Figure 2N and not shown), [34]. We next generated whole mount preparations of lateral wall that allow the visualization of the entire SVZ [35], [36]. Samples stained for YFP and GFAP, were analyzed by confocal microscopy. Several GFAP/YFP double positive cells, displaying long cellular bundles –i.e. possibly belonging to the type B cell population [37], [38]-, were detected within the SVZ (Figure 2O). In addition, double positive cells were often placed around clusters of YFP^+^ cells displaying the bipolar morphology of migrating neuroblasts (Figure 2O).

Long term descendants expressing YFP, were also found within the SVZ of P90 double transgenic brains. Although their number was slightly reduced at this stage, YFP^+^ cells co-expressed markers of neural progenitor cells such as: GFAP, Olig2 and PSA-NCAM (Figure S2A-C).

### AspM progeny gives rise to neurons, astrocytes and oligodendrocytes of the post-natal brain

**Figure 3:**
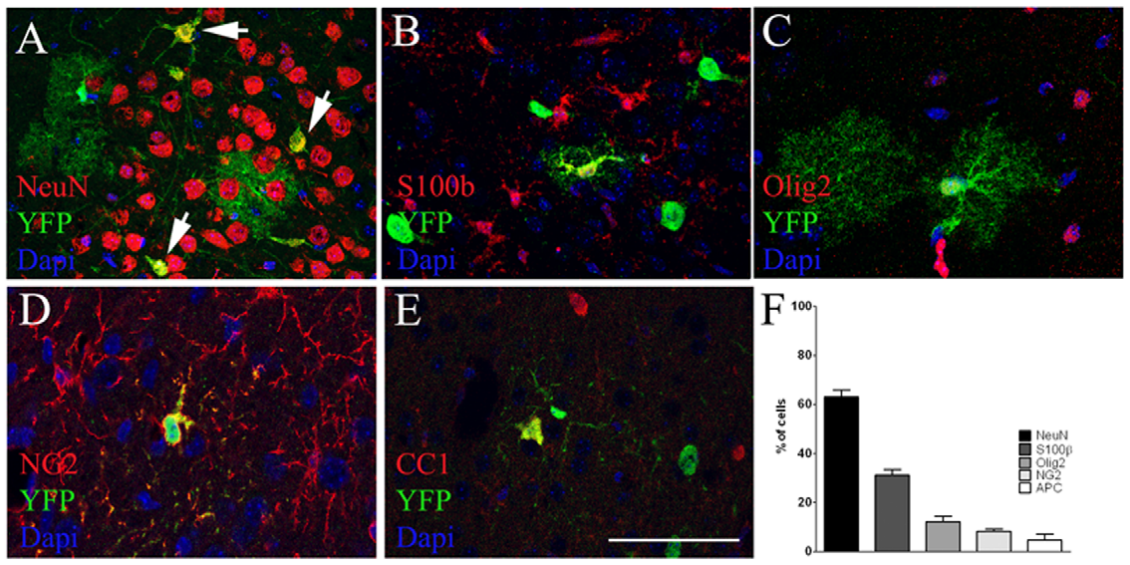
Embryonic AspM descendants give raise to neuronal and glial cell lineages in the adult brain. P30 coronal sections of AspM-CreER^T2^/Rosa26YFP double transgenic mice (n = 4) injected with Tam at the embryonic stages of E12.7 and E13.2 were assayed for YFP detection in the cerebral cortex. Cell counts were performed in a region encompassing the anterior bregma +1.2 and the posterior bregma −0.5. 63.1% (±2.8) of YFP^+^ cells in the cortex expressed NeuN (A, F), 31.3% (±2.1) expressed S100β (B, F), 12.2% (±0.8) Olig2 (C, F), 8.3% (±0.8) NG2 (D, F) and 4.7% (±2.5) CC1 (E, F). Percentages (±S.D.) are show in histogram plotted in panel F. Scale bar 100 µm.

To assess the multipotency of early AspM expressing cells, we next examined the YFP^+^ progeny, descending from E12.7/E13.2-primed AspM^+^ precursors in the cerebral cortex of P30 double transgenic brains. 63.1% (±2.8) of YFP^+^ cells expressed the neuronal marker NeuN (Figure 3A), [39]. As expected, the vast majority of these neurons occupied deeper regions of the cortical plate (not shown). We found also that 31.3% (±2.1) of YFP^+^ cells co-expressed the astroglial cell lineage marker S100β (Figure 3B), [40] and markers of the oligodendrocyte cell lineage, such as: Olig2 (12.2±0.8%) [41], NG2 (8.3±0.8%) [42] and APC (CC-1) (4.7±2.5%) (Figure 3C–F), [43]. Thus, early AspM expressing cells supply the adult brain of neurons, astrocytes and cells belonging to the oligodendrocyte lineage.

### AspM descendants located in the adult SVZ maintain features of neural progenitor cells

**Figure 4:**
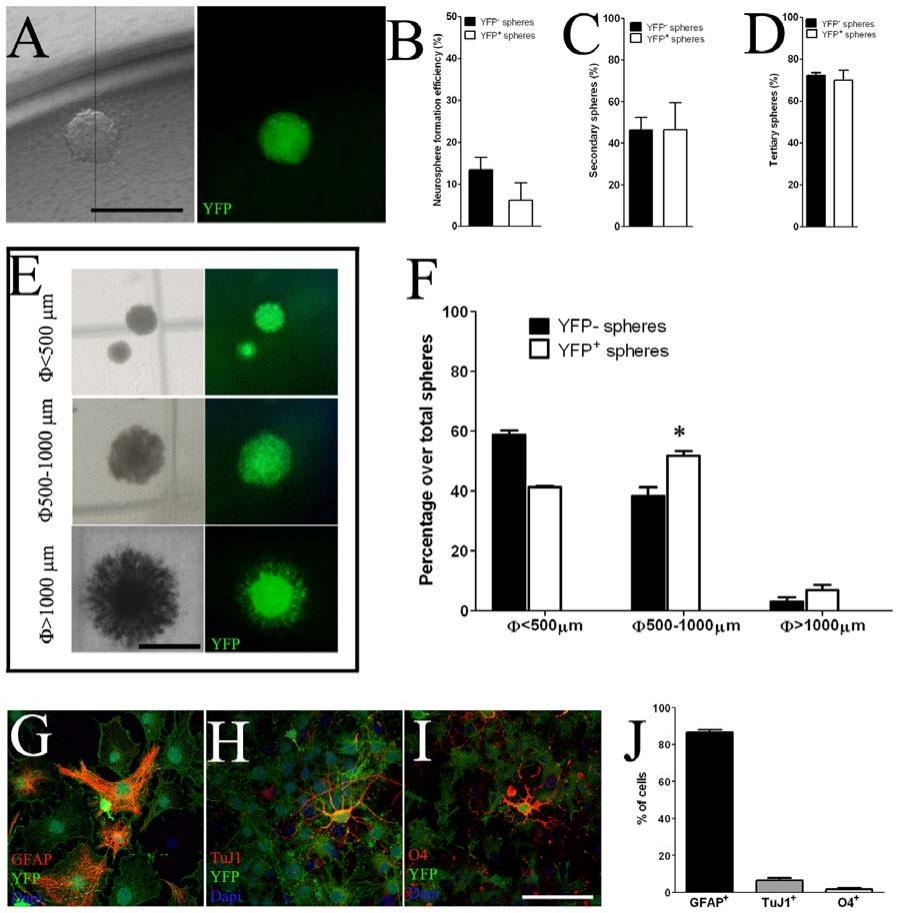
Embryonic AspM^+^ precursors give rise to aNSCs. Panel A shows single neurospheres derived from YFP^+^ cells. Quantification of the clonal efficiency of YFP^+^ cells was obtained by plating single neural stem cells on 96 wells (n = 3 independent experiments). Percentages (± S.E.M.) of YFP^−^ and YFP^+^ primary, secondary and tertiary neurospheres are plotted in panels B–D, respectively. Panel E shows representative images of YFP^+^ neurospheres of different sizes in the NCFC assay (<500 µm, 500–1000 µm and >1000 µm). AspM-CreER^T2^/Rosa26-YFP SVZ derived neural stem cells (n = 2 independent cell cultures) were propagated in vitro and then (after the 4^th^ IVP) assayed by NCFC assay. The mean percentages (± S.E.M.) of both YFP^−^ and YFP^+^ neurospheres of each size (n = 3 independent experiments for each cell culture) are plotted in panel F. Dissociated YFP^+^ neurospheres were capable to differentiate into astrocytes (G), neurons (H) and oligodendrocytes (I). Percentages (± S.D.) of each cell type are indicated in histogram on panel J. Scale bars: 1000 µm (panels A and E); 200 µm panels G–I. * <0.05, t-student.

To determine whether AspM-CreER^T2^ long term descendants that are detected in the adult SVZ, display features of neural progenitor cells, we tested their multipotency and self renewal capability by using standard clonal colony formation assay [44], [45]. AspMCreER^T2^/Rosa26-YFP double transgenic mice were injected with Tam at E12.5 and E13.5, cells were then collected from lateral ventricles of P30 brains and primary neural stem cell cultures were established [46]. Robust YFP expression levels were detected in many cells of SVZ-derived neurospheres, over serial *in vitro* passages (IVP) (not shown). Starting from the IVP-4, neurospheres cultures were dissociated in single cells and used in a standard clonogenic assay [4], [44]. Because each neurosphere was derived from a single cells, spheres were either entirely positive or negative for YFP (Figure 4A). In this assay, both single YFP^+^ and YFP^−^ cells give raise to primary, secondary and tertiary neurospheres with similar frequencies (Figure 4B, C). Furthermore, dissociated YFP^+^ cells from tertiary neurospheres were propagated in vitro for more than 10 IVPs, maintaining features of neural stem cell cultures (not shown). Starting from in IVP-5, dissociated cells are also capable to generate neurospheres of different dimensions, in a size based Neural Colony Forming Cells (NCFC) assay [45]. In this assay, dissociated cells were plated in a mitotic-containing collagen matrix and maintained in vitro for 3 weeks before assaying colony sizes. YFP^+^ cells give raise to neurospheres of different size, and 6.8% (±1.8) of them generated neurospheres of >1 mm in diameter, that presumably derive from *bona fide* NSCs (Figure 4E, F). Moreover, three independent large YFP^+^ neurospheres were dissected from plates, treated with collagenase before plating, and then propagated as YFP^+^ neurosphere cultures over serial IVPs (not shown). Dissociated YFP^+^ neurospheres were also assayed for multi-potency *in-vitro*. YFP^+^ cells, derived from large neurospheres, propagated for five IVPs, were then plated on matrigel coated dishes and kept in culture for seven days without growth factors. Differentiated YFP^+^ cells produced GFAP^+^ astrocytes (85.1±5.6%, Figure 4G, J), TuJ1^+^ neurons (6.5±1.2%, Figure 4H, J), and O4^+^ oligodendrocytes (1.7±0.7%, Figure 4I, J). Taken together, these results suggest that YFP^+^ cells descending from E12.5/E13.5 embryonic *AspM^+^* forerunners and derived from the P30 SVZ, maintain *in-vitro* molecular features of *bona fide* neural progenitor cells of the SVZ.

### Nestin-GFP^flox^-TK transgenic mice express the TK gene in RG cells of the developing forebrain and in aNSCs of the SVZ

In order to establish the functional role of forebrain *AspM*-expressing cells, we generated a further transgenic mouse line in which a floxed GFP gene was cloned downstream *Nestin* regulatory regions [47] and upstream to the suicide *Thymidine Kinase* (TK) gene (Figure S3A). Nestin-GFP^flox^-TK mice were characterized for the presence of GFP^+^ cells in both developing and post natal brains. During forebrain development, GFP-expressing cells were preferentially placed in the VZ/SVZ of the cortical field, always displaying the morphology of RG cells (Figure S3B). Proper expression of the Nestin-GFP^flox^-TK cassette was assessed during forebrain development, by comparing GFP and *Nestin* expression patterns on coronal section of E12.5 and P0 brains (Figure S3C, D). Virtually all GFP^+^ cells of E12.5 and P0 brains co-expressed *Nestin*, thus suggesting that the expression pattern of GFP fully overlaps endogenous *Nestin*. The presence and the distribution of GFP-expressing cells were also assayed in the post natal brain. P30 brain coronal sections, displayed many GFP/*Nestin* double positive cells in the SVZ (Figure S3E). Some of them, expressing the GFP at high levels, were also positive for the neural stem cells marker *Id1* [48] (Figure S3F). *Nestin*-expressing cells belong to the slow dividing cell population which displays a slow cell turnover. Accordingly, very few GFP^+^ cells of the SVZ incorporated the S-phase tracer EdU, after acute administration (10 hours), (Figure S3G). Altogether, these results confirm that Nestin-GFP^flox^-TK transgenic mice express both GFP and TK genes in RG cells of the developing brain and in slow dividing cells of the adult SVZ. By crossing these mice with AspM-CreER^T2^ mice, we could now kill virtually all AspM/Nestin expressing cells of the developing forebrain.

### Selective killing of AspM-CreER^T2^/Nestin-GFP^flox^-TK cells severely impairs forebrain development and Vz/SVZ cell proliferation

**Figure 5:**
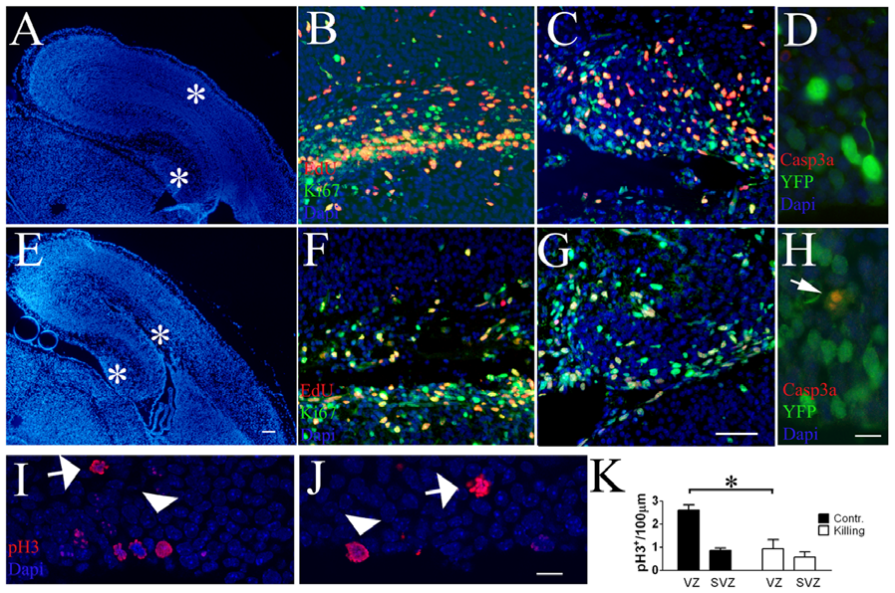
The selective killing of E12.7 AspM+ cells impairs neurogenesis. AspM-CreER^T2^/Nestin-GFP^flox^-TK double transgenic mice (n = 4, E–G) and their control litters –i.e. mice lacking the Nestin-GFP^flox^-TK transgene- (n = 4, A–C) were injected with Tam at E12.7 and E13.2, then daily administered with GCV and treated with EdU one hour before the sacrifice at E18.5. Coronal sections of double transgenic mice show a substantial derangement of brain architecture as shown in panels A and E. AspM-CreER^T2^/Nestin-GFP^flox^-TK brains were stained for EdU and Ki67 detection. Double transgenic mice administered with Tam/GCV show a significant reduction of proliferating cells within the cortical VZ/SVZ (F) and in the dentate gyrus (G), when compared with their controls (B, C). Sections from controls (I) and double transgenic mice (J) were stained for pH3 detection, and the mean number of cells/100 µm (± S.D.) is plotted on panel K. AspM-CreER^T2^/Nestin-GFP^flox^-TK/Rosa26YFP transgenic mice and their control litters (AspM-CreER^T^/Rosa26YFP mice) were administered with Tam and GCV (n = 4 for each group). Brains were collected at E16.5 and probed for YFP and Casp3a. Double positive cells are detected in triple transgenic mice (arrow in H indicates a double positive cells in the VZ of triple transgenic mice), but not in their controls (D). * p<0.05, t-student. Scale bar 100 µm and 10 µm in panels G and H, respectively.

**Figure 6:**
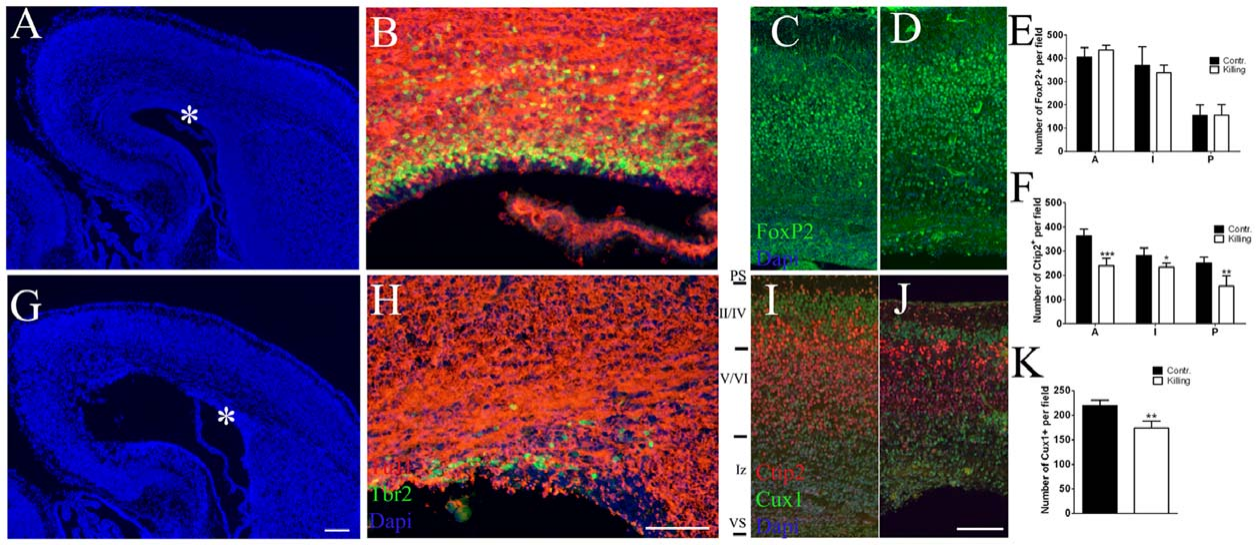
The selective killing of E12.7/E13.2 AspM+ cells altered the forebrain cortical organization. Panels A, G show low magnification of E18.5 coronal sections from a control –i.e. embryos lacking the Nestin-GFP^flox^-TK allele (A) and AspM-CreER^T2^/Nestin-GFP^flox^-TK (G) forebrains both treated with Tam and GCV (n = 3 for each group). Asterisks indicate the VZ/SVZ regions assayed for Tbr2 and TuJ1 detection. Control mice displayed a distinct layer of Tbr2^+^ BP cells that was clustered in the outer VZ (B). In contrast, double transgenic mice treated with GCV displayed a severe reduction of these cells (H). Double transgenic mice show a preserved cortical layer organization, although upper layers appeared thinner than normal. Coronal sections from control (C) and double transgenic (D) brains stained with Foxp2, which label deep layer neurons, show the preservation of deep cortical layer. Cells were count at three different levels along the anterior-posterior axes and the mean number of FoxP2^+^ cells (± S.D.) is plotted on panel E. Sections were also stained for Ctip2 (layer V) and Cux1 (layer II–IV) in control (I) and double transgenic (J) brains. Cell counts are provided in panels F and K, and show a significant reduction of upper cortical neurons in AspM-CreER^T2^/Nestin-GFP^flox^-TK mice. VS, ventricular surface, Iz intermediate zone, PS pial surface. * p<0.05, ** p<0.01, *** p<0.001, t-student. Scale bar 100 µm.

The activation of the TK gene was induced in AspM-CreER^T2^/Nestin-GFP^flox^-TK mice by injecting Tam at days E12.7 and E13.2. Selective killing of AspM/Nestin^+^ cells was obtained by treating mice with Gancyclovir (GCV) from E14.5 until E18.5. One hour before the sacrifice, at E18.5, mice received the EdU tracer to study proliferating cells of the brain. Double transgenic brains injected with Tam/GCV were significantly reduced in their size, when compared with control embryos (Figure 5A, D; Figure 6A, C). In particular, the dimensions of dentate gyrus and cortical wall were severely reduced (Figure 5A, D). The selective killing of E12.5/E13.2 AspM/Nestin^+^ forerunners affected cell proliferation in the cortical wall (Figure 5B, E) and the hippocampus (Figure 5C, F) of E18.5 embryos, as measured by Ki67^+^ and EdU^+^ staining. In addition, pH3^+^ mitosis placed at the ventricular lining were significantly reduced in double transgenic mice treated with Tam/GCV (Figure 5I–K). TK/GCV paradigm has been successfully used in tumor cells and graft versus host disease [49]. However, the cytotoxic effects mediated by GCV administration might be extended also to TK untransduced cells –i.e. the bystander effect- [50]. To assess the specificity of GCV-mediated cell death occurring in Tam/GCV treated embryos, we generated AspM-CreER^T2^/Nestin-GFP^flox^-TK/Rosa26YFP transgenic mice. Triple transgenic mice were firstly injected with Tam at E12.7/E13.2 and then treated with GCV at E14.5 and E15.5. Mice were sacrificed at E16.5 for the detection of both the activated Caspase 3 (Casp3a) and the YFP markers. Very few Casp3a^+^ cells were detected in control mice –i.e. embryos lacking the Nestin-GFP^flox^-TK allele (Figure 5D) but receiving GCV-. On the other hand, the analysis of triple transgenic mice showed that the number of Casp3a^+^ cells specifically increased in VZ-confined YFP expressing cells (Figure 5H). Although we cannot exclude that some YFP^−^ cells undergo to cell death during GCV treatment, these results suggest that the vast majority of dying cells belong to YFP^+^ cell population that expresses both AspM-CreER^T2^ and Nestin-GFP^flox^-TK transgenes.

### Selective killing of AspM-CreER^T2^/Nestin-GFP^flox^-TK cells severely impairs laminar organization of the cortex

The reduction of VZ proliferating cells occurring upon Tam/GCV treatments was also accompanied to a significant reduction of *Tbr2* expressing cells –i.e. BP cells- in E18.5 double transgenic mice (Figure 6B and D). Alterations of cell proliferation in germinal niches of the developing brain can also impact on cortical lamination. We therefore looked for changes in the cell distribution and neuronal density of cortical layers in E18.5 Tam/GCV treated AspM-CreER^T2^/Nestin-GFP^flox^-TK brains. We performed the immuno detection of FoxP2, which is predominantly expressed by early born deep-layer neurons [51], in both transgenic and control mice. The total number of FoxP2^+^ cells, however, did not change between double transgenic and control mice treated with Tam/GCV, as well as their distribution within the cortical wall (Figure 6C–E). We next extended our investigation to other cortical layers by staining sections for Ctip2 and Cux1 which are expressed in layers V [52] and layers II–IV [53] respectively (Figure 6F–K). Double transgenic mice showed consistent thinning of upper cortical layers that was accompanied by a preferential reduction of superficial later-born neurons, as demonstrated by a significant reduction of Ctip2^+^ and upper Cux1^+^cells (Figure 6F, K). Thus, starting from E14.5 the selective killing of AspM-CreER^T2^/Nestin-GFP^flox^-TK expressing cells impaired neurogenesis and reduced the number of neurons of upper cortical layers.

### AspM expressing cells of the SVZ are proliferating progenitors that generate GCL neurons

**Figure 7:**
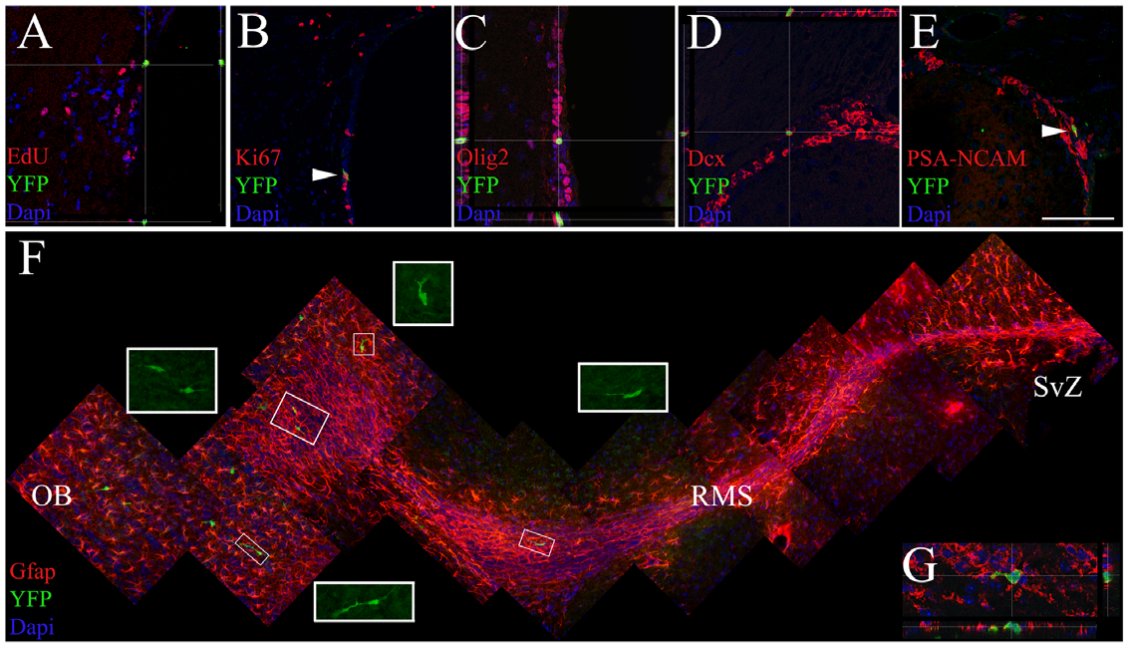
*AspM* is expressed cells of the adult SVZ sharing feature of progenitor cells. P30 AspM-CreER^T2^/Rosa26YFP mice (n = 4) were injected with Tam for 5 days. Mice were subsequently injected with EdU for further 5 days and then sacrificed. YFP^+^ cells were exclusively placed in the SVZ, as shown by confocal sectioning (A–E). YFP^+^ cells incorporated the EdU S-phase tracer (A), co-expressed Ki67 (B), Olig2 (C), Dcx (D) and PSA-NCAM (E). Scale bar 100 µm. Double transgenic mice (n = 4) injected with Tam as above, were also collected 17 days after the last Tam injection. Sagittal brain sections were probed for the detection of YFP^+^ cells along RMS and in OB. The position of the RMS, in each brain, was identified by staining slices for GFAP (F). The vast majority of YFP^+^ cells were located along RMS (F). Moreover, these cells displayed cellular processes which are typically of migrating neuroblasts (insets in F show high magnification of YFP^+^ cells detected in the RMS). Panel G shows a migrating YFP^+^ cell along the RMS, co-expressing PSA-NCAM. Scale bar100 µm.

We next investigated whether post-natal SVZ-restricted *AspM* expressing cells (Figure S1D) do functions as neural progenitor cells. Starting from P30, AspM-CreER^T2^/Rosa26-YFP mice were injected with Tam for 5 days and then sacrificed 6 days after the last injection. Several YFP^+^ cells, were detected along the SVZ, some of them incorporated the S-phase tracer EdU (Figure 7A), and were Ki67^+^ (Figure 7B). YFP^+^ cells of the dorsal SVZ co-expressed GFAP (not shown), and markers of neural progenitor cells, such as: Olig2 (Figure 7C), Dcx (Figure 7D) and PSA-NCAM (Figure 7E) [54]. We next tested whether AspM^+^ cells of the adult SVZ retain the capacity to generate neuroblasts migrating to the olfactory bulbs (OB) [55]. AspM-CreER^T2^/Rosa26-YFP mice were injected with Tam for 5 days as above, and sacrificed 17 days after the last Tam injection. In this experimental paradigm, the vast majority of YFP^+^ cells were detected at the end terminal of the rostral migratory stream (RMS) (Figure 7F). These cells exhibited the morphology of migrating neuroblasts and expressed the PSA-NCAM marker (Figure 7F, G). A restricted number of them reached the OB granular region, and displayed the morphology of differentiated neurons (not shown).

**Figure 8:**
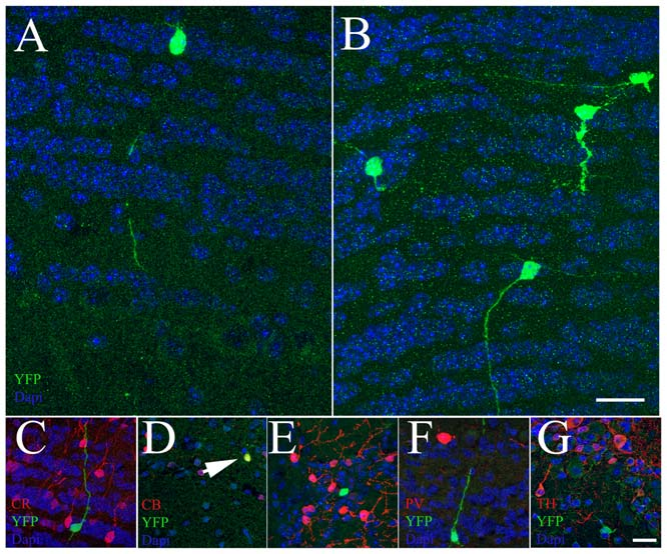
SVZ confined AspM+ cells give raise to granular OB neurons. P30 AspM-CreER^T2^/Rosa26YFP mice (n = 6 for each group) were injected with Tam for 5 days. YFP expressing cells were scored within the OB of mice that were sacrificed after 30 (A) and 60 (B) days from the last Tam injection. YFP^+^ cells were mainly placed within the GCL of OB. However, some of them were also detected within the internal plexiform layers and very few in the GL. Sections from brains collected 60 days after the last Tam injection, were also double labeled for YFP (C–G) and CR (C), CB (D, E), PV (F) and TH (G). Among the total number of YFP^+^ cells placed in OB, only a small number of them co-expressed CB (arrow in panel D). Scale bar 20 µm.

The vast majority of SVZ-derived precursors migrate along RMS to OB, where they differentiate into local interneurons of the granular (GCL) and glomerular layers (GL) [56], [57]. Periglomerular neurons are an heterogeneous cell population because these cells express different neurochemical markers and calcium binding proteins [58], [59], whereas interneurons in the GCL are an homogeneous population of GABAergic cells [58]. P30 AspM-CreER^T2^/Rosa26-YFP mice were injected with Tam as above and brains were collected after 30 and 60 days from the last Tam injection. To examine the neurochemical phenotype of YFP^+^ cells, OB coronal sections were labeled for Calretinin (CR), Calbinding (CB), Paravalbumin (PV) and Tyrosine hydroxylase (TH) [60], [61]. Thirty days after the last Tam injection, the vast majority of YFP^+^ cells (n = 320 cells) were placed within GCL and displayed the morphological phenotype of granular cells (Figure 8A). None of them, however, co-expressed CR, CB, PV or TH markers (not shown). Accordingly, the GCL contained the vast majority of YFP^+^ cells (n = 465 cells), also in mice collected after 60 days from the last Tam injection (Figure 8B). Nevertheless, 1.2 (±0.5)% of YFP^+^ cells co-expressed CB (Figure 8D), but none of them co-expressed CR, PV or TH markers (Figure8 C, F and G). Altogether these results suggest that AspM expressing cells of the adult SVZ differentially contribute to specific classes of OB neurons. While very few AspM descendants give raise to CB^+^ cells, the vast majority of them contribute to the generation of GCL neurons.

### AspM expression increased in growing neurospheres

We next examined whether post natal SVZ-derived NSCs maintain *in vitro* the expression of *AspM* after extensive passages. Neurospheres cultures were established from P30 SVZs and propagated for 20 IVPs. Single cells derived from these long lasting cultures were plated in neurospheres culture medium at the density of 8000 cells/mm^2^. Cells were then collected every day for the detection of *AspM* by real time PCR. Proliferating cells increased the size of neurospheres and rapidly increased *AspM* levels, that peaked at day 4 (Figure S4A). However, when spheres reached their maximum dimensions, these levels rapidly declined (Figure S4A). We next tested *AspM* expression in differentiating cultures. Cells were plated in the absence of growth factors and sampled each day to assess *AspM* expression levels. Accordingly to previous results[21], *AspM* expression rapidly dropped out in differentiating cell cultures (Figure S4B).

In conclusion, our data indicate that *AspM*-expressing cells of the developing forebrain supply of proliferating cells the germinal niches of both developing and post-natal brains. Furthermore, our long term fate mapping revealed that early *AspM* expressing cells can also differentiate in neurons, astrocytes and oligodendrocytes of the post natal brain. Supporting the notion that *AspM* expression is maintained by highly hierarchical progenitors of the brain.

## Discussion

The mechanisms by which proliferating cells of the developing brain are maintained within germinal niches of developing and post natal brains are largely unknown. Although, many experimental evidence supports the notion that adult neurons and glial cells derive from early neuroepithelial cells of the neural plate [62], [63], the precise identification of SVZ progenitors forerunners remains elusive [1]. Before the onset of neurogenesis neuroepithelial cells acquire new molecular features and become RG cells [63]. During early neurogenesis, these cells preferentially do symmetric division to ensure the expansion the cortical anlage [64], [65]. However, at later time points of the neurogenesis, dividing RG cells increase the rate of asymmetric cell divisions to generate neurons of the cortical plate [30]. Nevertheless, a subset of them is maintained into germinal niches of the brain to supply the adult SVZ of proliferating progenitor cells [1], [66]. Thus, during brain development the balance between RGs self renewal and the generation of committed descendants –i.e. progenitors fated to differentiate in neurons and in glial cells-, is crucial to ensure the appropriate size of brain and to maintain proliferating progenitor in germinal niches. Accordingly to this view, several genes controlling cell division/differentiation have been involved to prevent either the aberrant growth of the cortical primordium or the depletion of long term proliferating cells [67], [68], [69].

The human ASPM product has been linked to this function, because mutations in its coding region lead to severe microcephaly [19]. On the other hand, aberrant ASPM expression is tightly associated with malignant progression of gliomas, and *in vitro* short interference of ASPM resulted in G1-phase cell cycle arrest of tumor cells [21]. Recently it has been also demonstrated that the inactivation of *AspM* in mice produces a significant reduction of brain size [22]. By performing a short-term fate mapping experiment on E11.5 AspMCreER^T2^/Rosa26YFP embryos, we demonstrated that early forebrain *AspM*-expressing cells are RG cells that preferentially do symmetric cell divisions in the VZ. To assess whether or not *AspM*-expressing cells can give raise to long lasting proliferating cells of the brain, we performed a series of long term fate mapping experiments. In these experiments, we analyzed a single cohort of YFP-expressing cells inducing a transient Cre-mediated recombination of the Rosa26YFP locus after the onset of neurogenesis [14]. Recombined cells were assayed for the expression of proliferation markers and for their capacity to incorporate S-phase tracer at different time points along neurogenesis and, above all, in post natal brains. Surprisingly, a substantial number of *AspM* descendants were maintained as proliferating cells in germinal niches of both developing and post natal brains. Within the adult SVZ, recombined cells derived from early AspM expressing cells start to express new molecular markers which are typically associated to SVZ restricted adult neural progenitor cells. However, SVZ progenitors display heterogeneous features in term of markers and proliferating features. Thus, we took advantage of using clonogenic assays to demonstrate, *in vitro*, self renewal features and multipotency of these recombined cells [4]. Two independent assays demonstrated that recombined cells obtained from the adult SVZ can generate neurospheres with the same efficiency of wild type progenitors. Once neurospheres cultures were established, recombined cells were propagated for many in vitro passages, and demonstrated their multi-potency *in vitro*, when growth factors were removed from these cultures [70]. These data reinforce the idea that RG cells can supply the adult SVZ of proliferating cells and extend previous findings showing that progenitor cells fated to occupy the SVZ, as proliferating cells, derived from end gestational RG cells [1], [11], [14].

Since recombined cells detected within the SVZ account for a relative small percentage of total recombined cells, we tested whether early *AspM* forerunners are multipotent also *in vivo*. Adult brain analysis of double mutant mice revealed that *AspM* descendants differentiated in neurons, astrocytes and oligodendrocytes, thus suggesting that also early *AspM*-expressing cells are multipotent progenitors.

To test the role of early *AspM*-expressing forerunners, we performed their selective killing by using a novel transgenic mouse line: Nestin-GFP^flox^-TK mice. These mice express the TK gene under the control of both *Nestin* promoter region [47] and a floxed GFP cassette. TK/GCV system may induce the killing of cells not expressing the TK transgene –i.e. the so called “bystander effect”[50]. To rule out this possibility we generated triple transgenic mice in which recombined cells were traced by Rosa26YFP allele. The analysis of these mice demonstrated that cell death mainly occurs in recombined cells, expressing all transgenes, of the germinal brain niches. Upon Cre mediated recombination, the excision of the GFP cassette allows the expression of the TK gene. Starting from E12.7/E13.2, we were able to kill a single cohort of Nestin/AspM-expressing cells, causing a severe depletion of proliferating cells in the end gestational brain and a significant reduction of upper cortical neurons. This result reinforced the idea that early *AspM*-expressing cells are high hierarchical progenitors fated to maintain a proliferating pool during neurogenesis. Indeed, the selective ablation of these cells caused the significant reduction of RG cells in end gestational brains

We next investigated whether SVZ restricted adult *AspM* expressing cells, retain features of multipotent cells, as we have found in embryonic *AspM* forerunners. To achieve this goal, we performed another series of fate mapping experiments on AspM-CreER^T2^/Rosa26-YFP mice, changing the paradigm of Tam administration. As we have demonstrated by radioactive in situ hybridization and immunofluorescence, *AspM* expressing cells are prevalently located at the ventricular lining. We firstly demonstrated that AspM expressing cells can also express the Cre recombinase and that a substantial number of them can recombine the Rosa26YFP locus. Thus, we firstly performed a short term fate mapping analysis of these cells to assess if they do functions of proliferating cells. Mice were administered with Tam at P30 for five days, and then a short washing out period was applied before assaying recombined cells for their capability to incorporate the S-phase tracer EdU. Of note, in the adult brain neither neurons nor glial cells of the parenchyma displayed any recombination of the Rosa26YFP locus. Indeed, YFP expressing cells were detected only within the adult SVZ and a significant number of them were proliferating cells. Since the vast majority of SVZ proliferating cells are fated to generate neurons of the olfactory bulbs [35], we extended our observations by performing a long term fate mapping experiments of SVZ recombined cells. By increasing the washing out period, we were able to trace SVZ descendants along the RMS. These cells expressed the type-a marker PSA-NCAM and properly reached the olfactory bulbs. Extending the washing out time we also assessed that adult AspM descendants preferentially give rise to GCL neurons. Indeed, very few YFP^+^ cells were detected within the GL and mitral layers. This result suggests that AspM expressing cells of the adult SVZ might be commitment to generate only a specific subset of OB neurons. However, it remains to elucidate whether this feature may reflect differences in the progenitor pools of SVZ cells. Indeed, our current study cannot provide a sufficient resolution to determine the mechanism/s of AspM differentiation in vivo and, above all, molecular mechanisms that drive the expression of AspM in neural progenitor cells.

## Materials and Methods

### Gene targeting and transgenic mouse lines

Mice were maintained in pathogen-free conditions at San Raffaele Hospital mouse facility (Milan, Italy). All efforts were made to minimize animal suffering and to reduce the number of mice used, in accordance with the European Communities Council Directive of November 24, 1986 (86/609/EEC). All animal experimental protocols were approved by the Ethics Review Committee for Animal Experimentation of the Italian Ministry of Health. Procedures were performed according to the guidelines of the Institutional Animal Care and Use Committee of the San Raffaele Scientific Institute (protocol number 329/2007).

AspM-CreER^T2^ transgenic mouse line was generated by using “*recombineering*” technology. Briefly, the BAC expressing CreER^T2^ gene [24] under the control of AspM regulatory regions was cloned by targeting the genomic region (approximately 140 kb) of the BAC RP-24-267-F8, which contains the entire locus and approximately 70 kb of its promoter region. A *frt* flanked Kanamycin gene was inserted downstream CreER^T2^ [24] for the selection in bacteria. Homologous recombination was obtained in EL250 *E. Coli* strain accordingly to “*recombineering*” protocols [71], (http://web.ncifcrf.gov/research/brb/recombineeringInformation.aspx). The CreER^T2^–*frt*-Kana-*frt* cassette was inserted in the first methionine of AspM gene. Upon recombination, recombined BAC was tested for proper insertion of CreER^T2^ gene by direct sequencing. The *frt* flanked Kanamycin gene was then removed by activating the expression of the inducible Flpe recombinase in EL250 bacteria. DNA was injected in FVB zygotes and then targeted zygotes were surgically implanted into foster mothers. Founders were backcrossed in C57/BL6J mouse strain. Fate mapping experiments were performed by crossing AspM-CreER^T2^ transgenic mice with Rosa26YFP transgenic mice (obtained from Jackson laboratory).

We also generated Nestin-GFP^flox^-TK transgenic mouse line by using lentiviral technology. We used a third generation sin-lentiviral vector [72] to generate *NestfloxGFPfloxTK* targeting lentivirus. A conserved 1.8 Kb second intronic region of the rat *Nestin* [73] gene was cut out (XbaI, HindIII) from the p401ZgII plasmid (a gift of Dr. McMahon, Harvard University, Cambridge, MA) and sub-cloned upstream to the minimal promoter of the Hsp68 gene. The *loxP* sites were produced synthetically and were cloned upstream and downstream to the EGFP coding region. EGFP was obtained from the BamHI-SalI fragment of the #277 PGK-GFP lentivirus construct (a gift of Dr. Naldini, San Raffaele Hospital, Milan, Italy). Downstream to the EGFP sequence we sub-cloned the suicide gene Thymidine Kinase. BamHI-XbaI fragment TK coding sequence was cut out from the pBSIISK-TK construct. Finally, we inserted the IRES-lacZ fragment obtained from the pMODLacZnls plasmid downstream the TK gene. Transfer vector and the packaging plasmids: pMDLg/pRRE, pRSV-REV and pMD2.VSVG were transfected into 293T cells [74] by using the calcium phosphate precipitation method. Then, 14–16 hours later the DNA transfection, medium was replaced and 36 hours later cells supernatants were collected and filtered through a 0.22-µm pore nitrocellulose filter. To obtain high titer vector stock, the cell supernatants were concentrated by ultracentrifugation (55K g, 140 min, 20°C). Then, supernatants were discarded and the pellets were suspended in 100 µl of PBS 1x, split in 20 µl aliquots and stored at −80°C. LV stock was titrated by infecting HeLa cells with serial dilution of the viral stock and flow cytometry assay. Nestin-GFP^flox^-TK founder mice were generated by the *NestfloxGFPfloxTK* vector injection into the perivitelline space of C57Bl/6 zygotes. Two independent lines (#7457 and #7454) were generated and characterized for the presence of one copy of the inserted transgene. Experiments were conducted on #7454 transgenic mouse line. Mice were genotyped by PCR using genomic DNA and the following primers: FW, 5′-AACTTTCCCCGGAGAGCATCCACGC, Rev1, 5′- TAGGTCAGGGTGGTCACGAGGGT, Rev2, TGTTGATGGCAGGGGTACGAAGC.

### Tamoxifen, Ganciclovir & EdU administrations

Tamoxifen (Sigma) was dissolved in EtOH/Sunflower oil 10%/90% and injected at the following concentration 160 mg/kg. For the embryonic induction of AspM-CreER^T2^/Rosa26YFP double transgenic mice, Tam was intraperitoneally injected into pregnant mice at embryonic days of E10.5//E11.5 and E11.5/E12.5 for short term fate mapping; E12.7 and E13.2 for long term fate mapping. For induction in adult mice, Tam was injected intraperitoneally at the same concentration, into 4 weeks old mice once a day for 5 consecutively days. Ganciclovir (GCV, Roche) was administered in pregnant females as single daily intraperitoneal injection at a dose of 100 mg/kg starting from E14.5 until E16.5 or E18.5. Cell proliferation was assayed in vivo, in adult and embryonic forebrains, injecting mice with the S-phase tracer 5-ethynyl-2′-deoxyuridine (EdU, Invitrogen) at the following concentration: 100 mg/kg.

### Immunofluorescence and in situ hybridization

Immunofluorescence was performed as previously described [75]. Briefly, embryonic brains were carefully dissected in cold PBS 1X and fixed 4% paraformaldehyde in PBS pH 7.2 and then cryoprotected for at least 24 h in 30% Sucrose (Sigma) in PBS at +4°C. Adult mice were killed at P30 by anesthetic overdose and transcardially perfused with 4% paraformaldehyde in PBS pH 7.2. Dissected brains were post-fixed in the same solution for 12 hours at +4°C and then cryoprotected as above described. Coronal brain sections (10 µm) were incubated with blocking solution (PBS 1x, FBS 10%, BSA 1 mg/ml and Triton 0.1%) for 1 hour and then primary antibodies were applied in the same solution over night at +4°C. The following primary antibodies were used: rabbit α-AspM (1∶500, Millipore); rabbit α-CR (1∶1000, Swant); rabbit α-CB (1∶1000, Swant); mouse α-PV (1∶1000, Abcam); rabbit α-TH (1∶200, SIC); rabbit α-Casp3a (1∶100, NEB); rabbit α-FoxP2 (1∶200, Chemicon); goat α-Cux1 (1∶300, Santa Cruz); rat α-Ctip2 (1∶200, Abcam); rabbit α-pH3 (1∶200, Millipore); mouse α-RC2 (1∶100, Hybridoma bank); α-Ki67 (1∶1000, Novocastra); rabbit α-Tbr2 (1∶500, Chemicon); mouse α-TuJ1 (1∶1000, Millipore); click-it EdU Alexafluor 595 Imaging kit; rabbit α-Olig2 (1∶400, Chemicon); goat α-Dcx (1∶100, Santa Cruz); mouse α-GFAP (1∶1000, Chemicon); rabbit α-GFAP (1∶1500, Dako); mouse α-O4 (1∶100, Millipore); mouse α-PSA-NCAM (1∶500, Millipore); chicken α-GFP (1∶1000, Abcam); mouse α-NeuN (1∶500, Millipore); rabbit α-S100β (1∶500, Sigma); rabbit α-NG2 (1∶200, Santa Cruz); mouse α-CC1 (1∶100, Calbiochem); mouse α-Nestin (1∶100, BD); rabbit α-GFP (1∶500, Molecular Probes); rabbit α-Id1 (1∶100, Biocheck ink). Appropriate fluorophore-conjugated (Alexa Fluor 488, 546 and 633, Molecular Probes) or secondary antibodies were used accordingly manufacturer's instructions. When signal amplification was necessary (Id1, S100β), biotinylated goat and rabbit secondary antibodies (1∶200, Vector laboratories) were used. The biotinylated secondary antibody was further reacted with avidin and biotinylated HRP complex (1∶250, TSA-fluorescent system Perkin Elmer). HRP activities were revealed with Tyramide-Alexa 488 or Tyramide-Cy5 (1∶100, TSA-fluorescent system Perkin Elmer). Omission of the primary antibodies showed no specific staining. Nuclei were stained with 4′-6-diamidino-2-phenylindole (DAPI, Roche). Whole mounts preparation of the lateral ventricles were obtained from P30 transgenic mice as previously described [75]. Briefly mice were killed and perfused with saline. Upon the hippocampus was discarded, brain was cut along the rostro-caudal axes and the SVZ was removed by microdissecting scissor. Dissected lateral hemispheres were fixed 3 hours in 4% paraformaldehyde on ice. Then, explants were washed three times in PBS 1X, 0.5% Triton X100. Explants were incubated in PBX1X, BSA 1 mg/ml, FBS 10%, Triton X 100 0.3% for 1 hour at +4°C. Primary antibodies were diluted in the same buffer and incubated over night a +4°C. The next day, explants were washed in PBS 1X Triton X-100 0.3% and proper secondary antibodies conjugated with alexafluor 488, 546 and 633 dyes were applied in PBX1X, BSA 1 mg/ml, FBS 10%, Triton X 100 0.3% for 3 hours at room temperature. After staining, these explants were further dissected to remove the neural parenchyma and mounted on coverslip. Confocal reconstructions of the SvZ were taken on Leica SP5 microscope.

Light (Olympus, BX51 equipped with 4× and 20× objectives) and confocal (Leica, SP5 equipped with 20× and 40× objectives) microscopy was performed to analyze tissue staining. Analyses were performed by using Leica LCS lite and Adobe Photoshop CS software. Cell counts were done in the cerebral cortex of E13.5, E15.5 and P0 brains. Cells were also counted in cortical fields of P30 brain in a region encompassing the anterior bregma +1.2 and the posterior bregma −0.5.

### In situ hybridization

In situ hybridization were performed as previously described [75], [76]. Briefly, 10 µm-thick brain sections were post-fixed 15 min in 4% paraformaldehyde, then washed three times in PBS. Slides were incubated in 0.5 mg/ml of Proteinase K (Roche) in 100 mM Tris-HCl (pH 8), 50 mM EDTA for 10 min at 30°C. This was followed by 15 min in 4% Paraformaldehyde. Slices were then washed three times in PBS then washed in H2O. Sections were incubated in triethanolamine (Merk) 0.1 M (pH 8) for 5 min, then 400 µl of acetic anhydride (Sigma) was added two times for 5 min each. Finally, sections were rinsed in H2O for 2 min and air-dried. Hybridization was performed overnight at 60°C with α-UTP-P^33^ (G.E.) riboprobes at a concentration ranging from 10^6^ to 10^7^ counts per minute (cpm). The following day, sections were rinsed in SSC 5 X for 5 min then washed in formamide 50% (Sigma)-SSC 2 X for 30 min at 60°C. Then slides were incubated in ribonuclease-A (Roche) 20 mg/ml in 0.5 M NaCl, 10 mM Tris-HCl (pH 8), 5 mM EDTA 30 min at 37°C. Sections were washed in Formamide 50% SSC 2X for 30 min at 60°C then slides were rinsed two times in SSC 2X. Finally, slides were dried by using ethanol series. Lm1 (G.E.) emulsion was applied in dark room, according manufacturer instructions. After 10 days, sections were developed in dark room, counterstained with Dapi and mounted with DPX (BDH) mounting solution. The following probes were used: mouse *AspM* riboprobe (a gift of Dr. Walsh, Beth Israel Deaconess Medical Center, Boston-USA) and *Cre* riboprobe that was generated by cloning the SalI-PstI fragment corresponding to the entire Cre cds, from pCDNA3-Cre vector into pBSII-SK vector. For Digoxigenin in situ hybridization was performed as previously described [77]. Microphotographs of sections were digitalized in dark field light microscopy (Olympus BX51, and 4× objective) by using a CCD camera (Leica). Images manipulations were performed by using Adobe Photoshop CS. To confirm the specificity of the different RNA probes, sense strand RNA probes (showing no signal) were used as negative controls.

### Laser capture microdissection & Real Time PCR

P30 mice were deeply anesthetized and then were rapidly perfused transcardially with RNase free 0.9% saline containing 10 U/ml of heparin. Brains were removed from the skulls, OCT embedded and rapidly frozen by immersion in liquid nitrogen. Laser mediated microdissections were obtained by using a Leica AS LMD equipment (objective 20×). Briefly, 25 µm coronal sections were generated starting from bregma +1.2 to bregma −0.5. Then, sections were dehydrated. From each slide both the SVZ and striatum were captured. An average of 55 (±10) dissections per mouse were collected and total RNA was extracted by using RNeasy Mini Kit (Qiagen) according to manufacturer's recommendations including DNase digestion. cDNA synthesis was performed by using ThermoScriptTM RT-PCR System (Invitrogen) and Random Hexamer (Invitrogen) according the manufacturer's instructions for standard PCR by using following primers: AspM f: GCCTCTCTCGGCCTCCATCAGCCTCCTTG AspM r: CAGGCAGCGTTGCTTCTATCAACA GCGTCG; Cre f: GCCTGCATTACCGGTCGATGCAACGA Cre r: GTGGCAGATGG CGCGGC AACACCATT. Gene expression analysis was performed by using the LightCyclerR 480 System (Roche) and SYBRR Green JumpStart™ Taq ReadyMix™ for High Throughput QPCR (Sigma). Each sample was normalized by using the housekeeping gene *Histone H3* with the following primers: H3 F: GGTGAAGAAACCTCATCGTTACAGGCCTGGTAC H3R: CTGCAAAGCAC CAATAGCTGCACTCTGGAAGC.

### Neural stem cell cultures generation and maintenance

Neural stem cell cultures were obtained from P30 AspM-CreER^T2^/Rosa26YFP mice primed with Tam at E12.7 and E13.2, as previously described [75]. Briefly, three mm-thick coronal sections were obtained from the anterior forebrain of P30 double transgenic mice (2 mm from the anterior pole of the brain). Dorsal SVZs were carefully dissected by using a fine scissor in the following dissociating medium: Earl's Balanced Salt Solution (Gibco) supplemented with 1 mg/ml Papain 27 U/mg; (Sigma), 0.2 mg/ml Cysteine (Sigma) and 0.2 mg/ml EDTA (Sigma). Then, dissected tissue was incubated in the same solution for 30 min at 37°C on a rocking platform. Finally, dissociated cells were plated in standard neuro-sphere growth medium Neurocult proliferation medium (Stem cell Technology) supplemented with EGF (20 ng/ml) and FGF2 (10 ng/ml). For each in vitro passage, single cells were obtained by incubating neurospeheres in Accumax (Sigma) for 10 minutes, then 8000 cells/mm^2^ were plated on T75 plastic flasks (Nunc). Neurospheres were propagated in vitro and assayed for self-renewal and cell differentiation after 4 in vitro passages (IVP), as previously described [46].

### Neurospheres formation assay

Neurospheres raised from AspM-CreER^T2^/Rosa26YFP neural stem cells (n = 2 independent cell cultures) after the 4^th^ IVP were dissociated in single cell suspensions with Accumax as above, and plated as single cells in a 96 wells multiwell. After 10 days, the number of both YFP^+^ and YFP^−^ neurospheres with a diameter >100 µm were counted in three independent experiments. To determine the cell renewal capacity of these cells, neurospheres were then dissociated and then re-cultured under the same condition as primary cultures. Again, ten days later, we determined the number of secondary neurospheres. This experiment was repeated for the detection of tertiary neurospheres. Primary, secondary and tertiary neurospheres were measured under inverted microscope equipped with fluorescence (Axiovert S100TV) and data were expressed as percentages ± S.E.M.

### Neural colony forming cell assay

AspM-CreER^T2^/Rosa26YFP neural stem cells (n = 2 independent cell cultures) at 5^th^ IVP were dissociated in single cells as above described, diluted in Neurocult proliferation medium at the concentration of 2.2×10^5^ cells/ml. Then, cells were mixed with collagen solution accordingly to manufacturer's instructions (Stem cell Technology) and approximately 2.500 cells were plated on 35 mm dishes. Dishes were incubated in a 100 mm petri dishes containing open 35 mm dishes filled with sterile water. Cultures were maintained at 37°C, 5%CO_2_ for 21 days and then visually assessed by using an inverted microscope equipped with fluorescence (Axiovert S100TV). Dishes were placed on a gridded scoring dish (Stem cell Technology) and scored at low magnification. Colony were classified into one of three categories: **a**, less then 0.5 mm diameter; **b**, 0.5–1 mm diameter; and **c**, more than 1 mm. Data were expressed as percentages ± S.E.M. of three independent experiments.

### Neural stem cell cultures differentiation assay

To analyze multipotency *in-vitro*, individual spheres (e.g., tertiary established neural stem cells cultures) were mechanically dissociated, and single cells plated onto Matrigel® (BD)-coated glass coverslips (12 mm diameter) in the presence of 20 ng/ml of FGF-2. After 72 hr in vitro, cells were shifted to *control medium* containing 1% fetal bovine serum (FBS) and 5 days later fixed in 4% paraformaldehyde pH 7.4 and processed for immune-fluorescence. Fixed cells were then rinsed with PBS 1x and incubated for 30 min at in PBS containing 10% normal goat serum (NGS)/0.1% Triton X-100. Cells were then incubate in primary antibodies for 2 hours. After washing, cells were incubated for 1 hour with the appropriate secondary antibodies. Samples were rinsed three times with PBS, counter stained with Dapi, and once with distilled water. Then slides were mounted with Fluorsave (Calbiochem). The following primary antibodies and antisera were used: rabbit anti- neuron specific tubulin type III (1∶1000, Tuj1, Covance), mouse α-O4 (1∶1000, Millipore), rabbit α-GFAP (1∶1000 Dako) and chicken α-GFP (1∶1000, Abcam). Appropriate fluorophore- (Alexa-fluor 488 and 546, Molecular Probes) conjugated secondary antibodies were used. The Samples were examined and photographed using a Olympus BX51, and 20× objective fluorescence microscope. The number of cells immunoreactive (IR) for different antigens was counted in n≥20 non overlapping fields per sample (up to a total of n≥1000 cells per sample) and data were expressed as mean percentage of immunoreactive cell (over total counted nuclei) ± S.D. from a total of n≥3 independent experiments.

## Supporting Information

Figure S1 **Design and activity of AspM-CreER^T2^ transgene.** Panel A shows the targeting construct containing the CreER^T2^ gene, the Kanamycin/Neomycin resistance cassette and the insertion point on *AspM* locus (top). In the bottom panel is shown the construct after the excision of resistance cassette. Panels B and C show forebrain coronal sections of E15.5 AspM-CreER^T2^ embryos probed for *AspM* (B) and *Cre* (C) detection, by radioactive in situ hybridization (n = 4). Panels D and E show coronal sections derived from P30 transgenic mice probed for *AspM* (D) and *Cre* (E) (n = 3). Panel F shows a representative P30 brain coronal section from AspM-CreER^T2^ mice (n = 3) that was used for laser capture microdissection of the SVZ and the striatum. Total mRNA was extracted from each pool of sections and was used for RT-PCR detection of *AspM* and *Cre* transcripts (F, right panel). AspM-CreER^T2^/Rosa26YFP mice were pulsed with Tam at E10.5/11.5 and collected at E12.5 (n = 4). Panel G shows the in situ hybridization for *AspM* coupled with the immuno-detection of YFP in a double transgenic brain. Arrows in panel G indicate double positive cells placed within the VZ. Panel H shows confocal sectioning of an adjacent section probed for AspM and YFP detection that confirmed the presence of double positive cells in germinal niches of AspM-CreER^T2^/Rosa26YFP brain. P30 AspM-CreER^T2^/Rosa26YFP mice (n = 3) injected with Tam for 5 consecutive days, then sectioned and probed for AspM and YFP detection. Arrow in panel I shows a double positive cells located at the ventricular lining of double transgenic mice. Scale bars 100 µm.
(TIF)

Figure S2 **Embryonic** ***AspM*** **precursors are maintained in the germinal niches of P90 brain.** AspM-CreER^T2^/Rosa26YFP mice administered with Tam at the embryonic stages of E12.7/E13.2 were collected at P90 (n = 3). Brain sections were probed for YFP and GFAP (A), Olig2 (B) and PSA NC AM (C). Confocal sectioning of the dorsal SVZ revealed the presence of scattered YFP/GFAP^+^ positive cells closely located at the ventricular lining (A). Adjacent sections revealed the presence of YFP cells co-expressing Olig2 (B) and PSA NCAM (C). Scale bar 100 µm.
(TIF)

Figure S3 **Design and activity of Nestin GFP^flox^-TK transgenic mouse line.** Panel A shows the Nestin GFP^flox^-TK construct carrying a floxed GFP and the *Tymidine Kinase* (TK) genes under the control of *Nestin* regulatory regions. Top panel indicates the construct expressing the *GFP* before the *Cre* mediated recombination. After *GFP* excision, the TK starts to be expressed in cells as shown in bottom panel. Panel B shows immune fluorescence for GFP in a E12.5 transgenic embryo (n = 4). GFP expressing cells in the cortical wall displayed the morphology of RG cells (insets in B). Panels C-E show double fluorescences for GFP and Nestin on coronal sections of E12.5 (C), P0 (D) and P30 (E) brains (n = 4 for each group). Virtually all GFP expressing cells of embryonic proliferating niches co-expressed Nestin. Accordingly, P30 coronal sections show GFP expressing cells of the lateral ventricles that co-expressed Nestin (arrowheads in E) and Id1 (arrows in F). P30 Nestin-GFP^flox^-TK transgenic mice were injected with EdU for 10 hours before sacrifice (n = 3). Very few GFP^+^ cells incorporated EdU (G) suggesting that they belong to the relatively quiescent adult neural stem cell population (e.g. type-B cells). Scale bar 100 µm.
(TIF)

Figure S4 **AspM expression is confined in proliferating neurospheres cultures.** SVZ derived neurospheres cultures (n = 3 independent cultures) were established from P30 brains. Cells were kept in culture for 20 IVPs, then single cells were plated at the concentration of 8000/cm^2^ in neurospheres cultures standard medium. Cells were collected for 6 consecutive days and *AspM* mRNAs levels were measured by real time PCR. Fold changes were calculated on the relative *AspM* expression measured at day 1. Mean values (±S.D.) derived from 3 independent experiments performed on these cultures are plotted on histogram in panel A. NSCs (n = 3 independent cultures) were plated on matrigel coated dishes and kept in culture without growth factors. Total RNA was collected for 4 consecutive days and *AspM* expression levels measured by real time PCR. Fold changes were calculated on the relative AspM expression measured on undifferentiated cells. Mean values (±S.D.) from 3 independent cultures are plotted in histogram of panel B. *** p<0.001, t-student.
(TIF)
