# Supplementary figures and images for: In Vivo Fate Analysis Reveals the Multipotent and Self-Renewal Features of Embryonic AspM Expressing Cells
Source: PLoS One. 2011 Apr 29;6(4):e19419. doi: 10.1371/journal.pone.0019419 (PMC3084851; doi:10.1371/journal.pone.0019419)

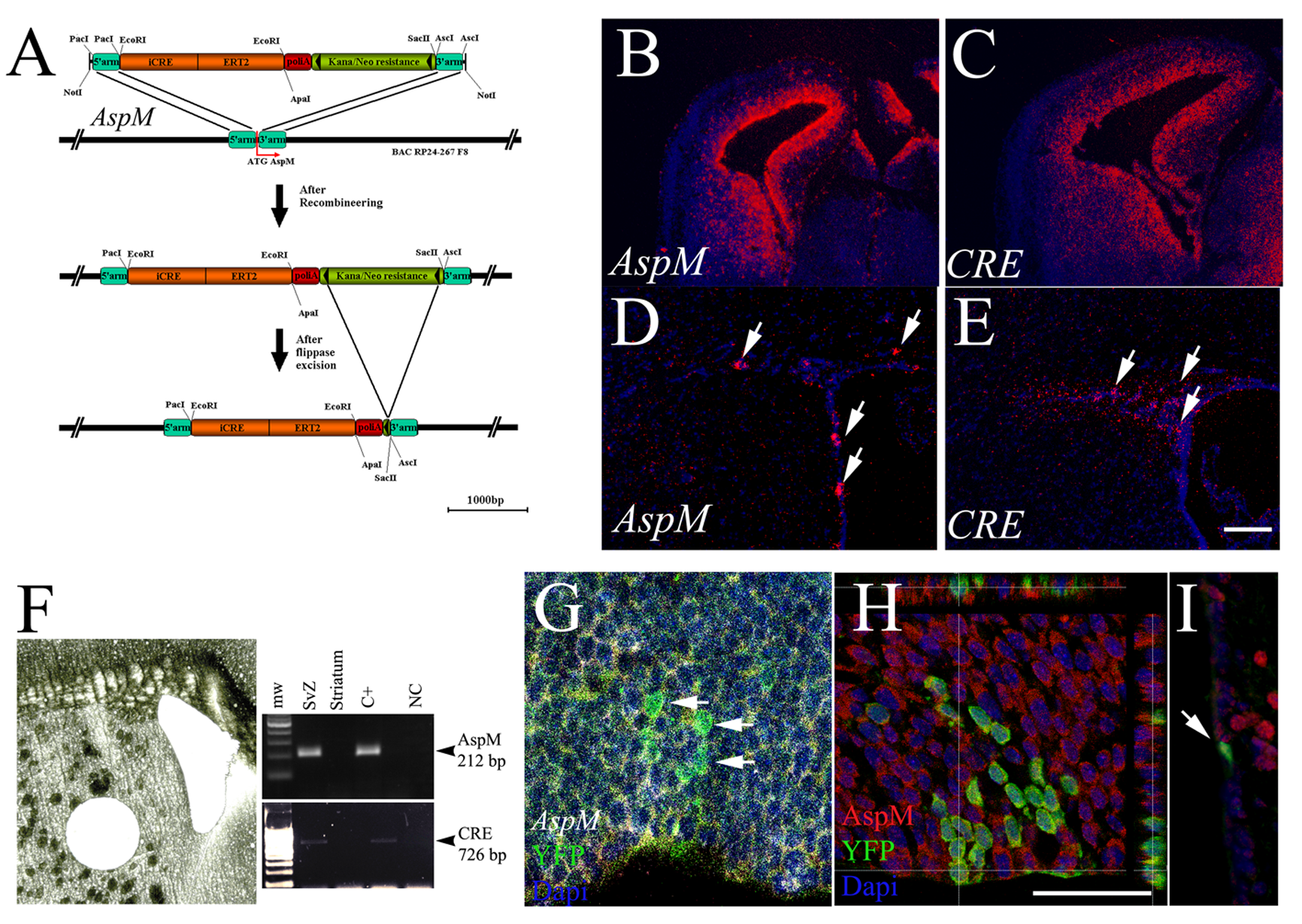

Supplement: Figure S1 — Design and activity of AspM-CreERT2 transgene. Panel A shows the targeting construct containing the CreERT2 gene, the Kanamycin/Neomycin resistance cassette and the insertion point on AspM locus (top). In the bottom panel is shown the construct after the excision of resistance cassette. Panels B and C show forebrain coronal sections of E15.5 AspM-CreERT2 embryos probed for AspM (B) and Cre (C) detection, by radioactive in situ hybridization (n = 4). Panels D and E show coronal sections derived from P30 transgenic mice probed for AspM (D) and Cre (E) (n = 3). Panel F shows a representative P30 brain coronal section from AspM-CreERT2 mice (n = 3) that was used for laser capture microdissection of the SVZ and the striatum. Total mRNA was extracted from each pool of sections and was used for RT-PCR detection of AspM and Cre transcripts (F, right panel). AspM-CreERT2/Rosa26YFP mice were pulsed with Tam at E10.5/11.5 and collected at E12.5 (n = 4). Panel G shows the in situ hybridization for AspM coupled with the immuno-detection of YFP in a double transgenic brain. Arrows in panel G indicate double positive cells placed within the VZ. Panel H shows confocal sectioning of an adjacent section probed for AspM and YFP detection that confirmed the presence of double positive cells in germinal niches of AspM-CreERT2/Rosa26YFP brain. P30 AspM-CreERT2/Rosa26YFP mice (n = 3) injected with Tam for 5 consecutive days, then sectioned and probed for AspM and YFP detection. Arrow in panel I shows a double positive cells located at the ventricular lining of double transgenic mice. Scale bars 100 µm. (TIF) [file pone.0019419.s001.tif]

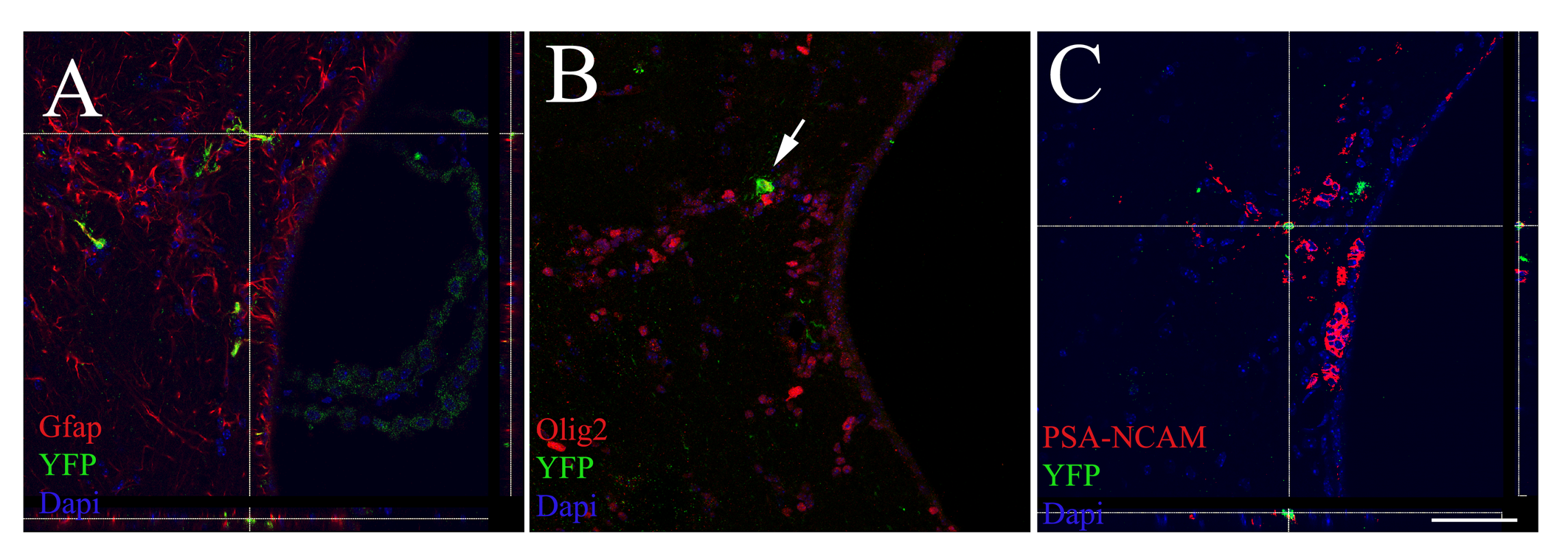

Supplement: Figure S2 — Embryonic AspM precursors are maintained in the germinal niches of P90 brain. AspM-CreERT2/Rosa26YFP mice administered with Tam at the embryonic stages of E12.7/E13.2 were collected at P90 (n = 3). Brain sections were probed for YFP and GFAP (A), Olig2 (B) and PSA NC AM (C). Confocal sectioning of the dorsal SVZ revealed the presence of scattered YFP/GFAP+ positive cells closely located at the ventricular lining (A). Adjacent sections revealed the presence of YFP cells co-expressing Olig2 (B) and PSA NCAM (C). Scale bar 100 µm. (TIF) [file pone.0019419.s002.tif]

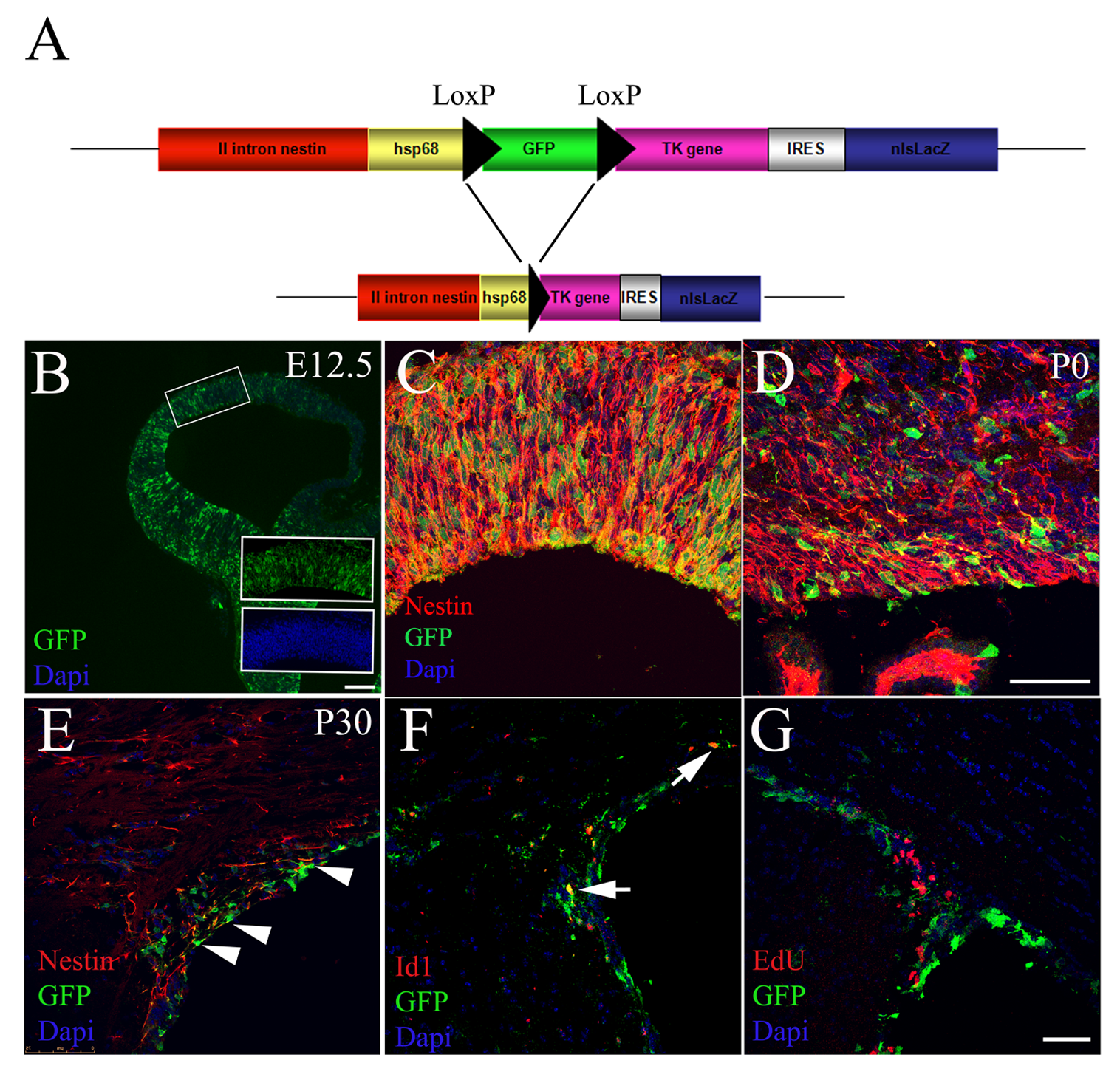

Supplement: Figure S3 — Design and activity of Nestin GFPflox-TK transgenic mouse line. Panel A shows the Nestin GFPflox-TK construct carrying a floxed GFP and the Tymidine Kinase (TK) genes under the control of Nestin regulatory regions. Top panel indicates the construct expressing the GFP before the Cre mediated recombination. After GFP excision, the TK starts to be expressed in cells as shown in bottom panel. Panel B shows immune fluorescence for GFP in a E12.5 transgenic embryo (n = 4). GFP expressing cells in the cortical wall displayed the morphology of RG cells (insets in B). Panels C-E show double fluorescences for GFP and Nestin on coronal sections of E12.5 (C), P0 (D) and P30 (E) brains (n = 4 for each group). Virtually all GFP expressing cells of embryonic proliferating niches co-expressed Nestin. Accordingly, P30 coronal sections show GFP expressing cells of the lateral ventricles that co-expressed Nestin (arrowheads in E) and Id1 (arrows in F). P30 Nestin-GFPflox-TK transgenic mice were injected with EdU for 10 hours before sacrifice (n = 3). Very few GFP+ cells incorporated EdU (G) suggesting that they belong to the relatively quiescent adult neural stem cell population (e.g. type-B cells). Scale bar 100 µm. (TIF) [file pone.0019419.s003.tif]

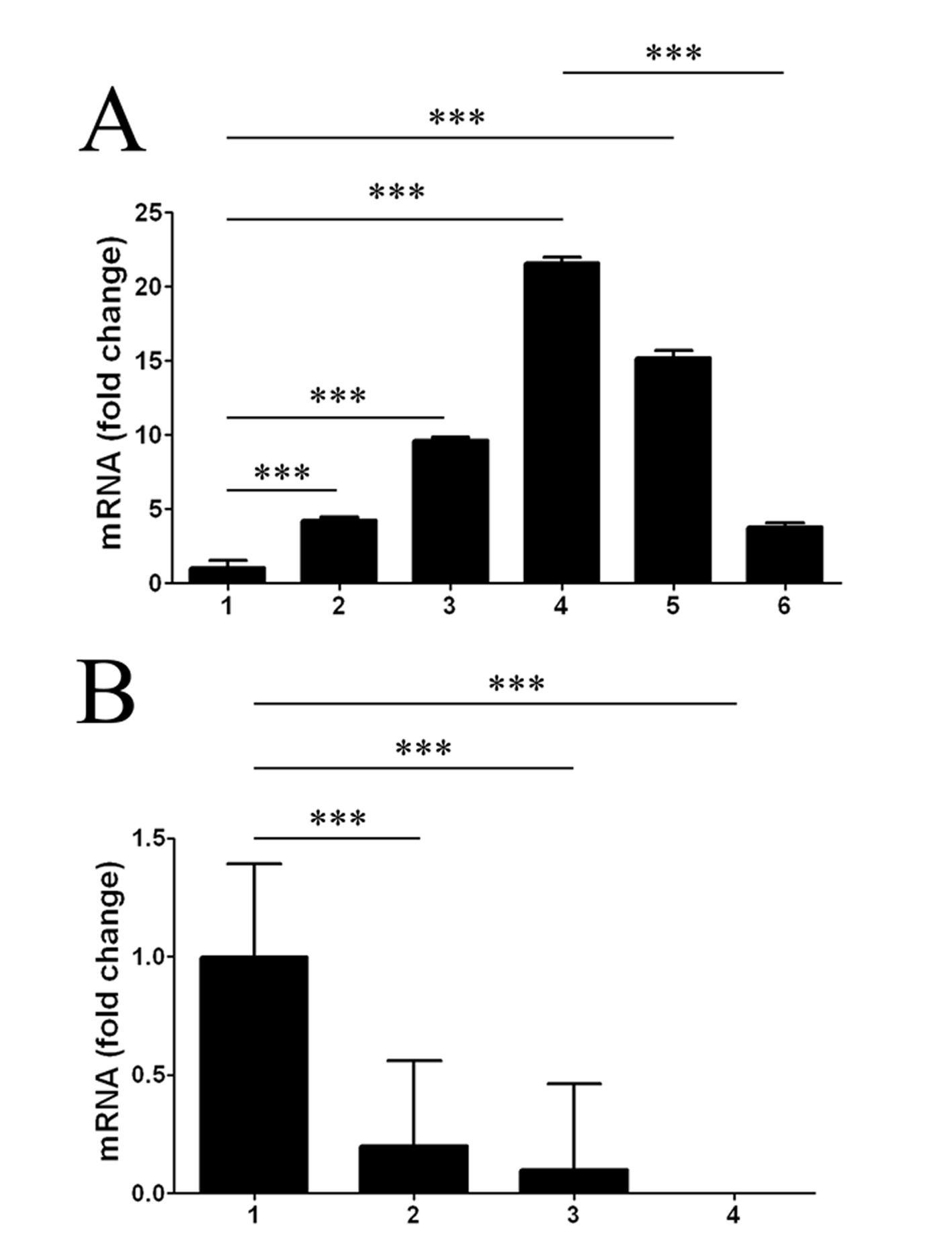

Supplement: Figure S4 — AspM expression is confined in proliferating neurospheres cultures. SVZ derived neurospheres cultures (n = 3 independent cultures) were established from P30 brains. Cells were kept in culture for 20 IVPs, then single cells were plated at the concentration of 8000/cm2 in neurospheres cultures standard medium. Cells were collected for 6 consecutive days and AspM mRNAs levels were measured by real time PCR. Fold changes were calculated on the relative AspM expression measured at day 1. Mean values (±S.D.) derived from 3 independent experiments performed on these cultures are plotted on histogram in panel A. NSCs (n = 3 independent cultures) were plated on matrigel coated dishes and kept in culture without growth factors. Total RNA was collected for 4 consecutive days and AspM expression levels measured by real time PCR. Fold changes were calculated on the relative AspM expression measured on undifferentiated cells. Mean values (±S.D.) from 3 independent cultures are plotted in histogram of panel B. *** p<0.001, t-student. (TIF) [file pone.0019419.s004.tif]
